# NGX6 gene mediated by promoter methylation as a potential molecular marker in colorectal cancer

**DOI:** 10.1186/1471-2407-10-160

**Published:** 2010-04-27

**Authors:** Minji Liu, Ya Peng, Xiaoyan Wang, Qin Guo, Shourong Shen, Guiyuan Li

**Affiliations:** 1 Department of Gastroenterology, The Third Affiliated Hospital of Xiang Ya School of Medicine, Central South University, Changsha 410013, Hunan, PR China; 2 Cancer Research Institute, Central South University, Changsha 410078, Hunan, PR China

## Abstract

**Background:**

Nasopharyngeal carcinoma associated gene 6 (NGX6) is down-regulated in most colon cancer cell lines and tumor tissues when compared with their normal tissue samples. As a novel suppress tumor gene, it could inhibit colon cancer cell growth and cell cycle progression. However, little is known about the transcriptional mechanisms controlling NGX6 gene expression. Recent findings suggest that epigenetic inactivation of multiple tumor suppressor genes plays an important role in the tumorigenesis of colorectal carcinoma (CRC). In this study, we explored the role of DNA methylation in regulation of NGX6 transcription.

**Methods:**

In the present study, we cloned the NGX6 promoter with characteristics of a CpG island by luciferase reporter assay. Then, the CpG methylation status around the NGX6 promoter region in colon cancer cell lines and colorectal tumor tissues was examined by methylation-specific PCR and bisulfite DNA sequencing. Finally, 5-Aza-2'-deoxycytidine (5-Aza-dC) treatment was used to confirm the correlation between NGX6 promoter methylation and its gene inactivation.

**Results:**

The sequence spanning positions -157 to +276 was identified as the NGX6 promoter, in which no canonical TATA boxes were found, while two CAAT boxes and GC boxes were discovered. Methylation status was observed more frequently in 40 colorectal cancer samples than in 40 adjacent normal mucosa samples (18/40 versus 7/40; P < 0.05). An analysis correlating gene methylation status with clinicopathological cancer features revealed that dense methylation of the NGX6 promoter was associated with colorectal cancer patients age (P < 0.05). Moreover, a trend was shown toward metastasis status and primary site in colorectal carcinomas with NGX6 promoter methylation (p = 0.056 and P = 0.067, respectively). In addition, 5-Aza-dC could induce NGX6 mRNA expression and NGX6 promoter demethylation in HT-29 cells.

**Conclusions:**

Down-regulation of NGX6 gene is related to the promoter methylation. DNA methylation of NGX6 promoter might be a potential molecular marker for diagnosis or prognosis, or serve as a therapeutic target.

## Backgroud

Colorectal cancer (CRC) is one of the most common neoplasms all over the world. In addition to multiple genetic alterations, it is now recognized that the development and progression of CRC is associated with epigenetic mechanisms, especially DNA methylation. The methylation of the cytosine residues in CpG-rich sequences (CpG island) located in the promoter regions of genes regulating cell proliferation, apoptosis, and DNA repair [1,2]. Determination of epigenetic events is a strong candidate for early detection of disease, since regulation of gene expression by aberrant DNA methylation is a well-characterized event in tumor biology, and is extensively described for CRC.

NGX6 is a novel EGF-like domain-containing gene identified by a location candidate cloning strategy [3]. Its mRNA expression level in nasopharyngeal carcinoma tissues was significantly lower than in normal nasopharyngeal epithelial tissues [4]. NGX6 was also down-regulated in colorectal carcinomas, and the frequency of down-regulation of NGX6 in colorectal carcinoma tissues with lymph node or distance metastasis (15/16) was significantly greater than in patients without metastasis (25/34) (P < 0.05) [5]. Indeed, over-expression of NGX6 gene in HT-29 cells can effectively inhibit cell growth and cell cycle progression from G1 to S phase by transcriptional regulation of some key cell cycle related genes [6]. Recent studies show that NGX6 gene can reduce tumor formation and tumor size in nude mice by down-regulating the EGFR/K-ras/JNK/c-Jun/cyclin D1 and Wnt/beta-catenin/TCF/LEF signal pathways [7-10]. Therefore, the loss of NGX6 function may be an important event in the progression of CRC and act as a novel candidate for tumor suppression. However, little is known about the down-regulation of NGX6 gene in CRC.

In the present study, we investigated whether the NGX6 gene in colorectal cancer was regulated by epigenetic mechanisms such as DNA methylation. Firstly, we cloned the NGX6 promoter with characteristics of a CpG island. Then, CpG methylation status around the NGX6 promoter region in colon cancer cell lines and tumor tissues was examined by methylation-specific PCR and bisulfite DNA sequencing. In order to demonstrate a functional association between NGX6 promoter methylation and its gene down-expression, we performed DNA demethylation analysis with colon cancer cell line HT-29 using methylation-specific PCR, RT-PCR and real-time PCR.

## Methods

### Cell lines and tumor tissues

Human colon carcinoma cell lines HT-29 and SW480 were from American Type Culture Collection (ATCC, Rockville, MD) cell bank. Cos7 cells were provided by the Cancer Research Institute, Xiangya School of Medicine, Central South University (Human, P.R. China). All cells were cultured in RPMI1640 medium containing 10% heat-inactivated fetal bovine serum (FBS) and incubated at 37°C in a humidified incubator containing 5% CO_2_.

Fresh colorectal cancer tissues and adjacent normal colorectal tissues were obtained from patients treated by primary surgery for colorectal cancer at Third Xiangya hospital surgery department (Hunan, People's Republic of China). All patients gave informed consent for the study to retain and analyze their tissue for research purposes. The samples were snap-frozen immediately following resection and stored in liquid nitrogen until processing. The 19 male and 21 female were aged from 18 to 81 years (mean 54.7 ± 15.1 years). For the colorectal portion, we obtained approval from the Ethic Committee of Central South University.

### Cloning and analysis of the NGX6 5'upstream regulatory region

The NGX6 promoter region in the 5' flanking region was predicted using the PromoterInspector and FirstEF programs. The CpG island was found using CpGplot of the European Molecular Biology Open Software Suite. To obtain the 5' flanking region of the NGX6 gene, PCR amplification on human genomic DNA (forward primer 5'-CGAGCCCAGAGGGTTTACTT-3', reverse primer 5'-GCCTCAATCTTCCCTGCTTC-3') was preformed in a 50-μl reaction mixture. After an initial denaturation step at 94°C for 10 min, the PCR reactions were carried out for 30 cycles at 94°C for 30 sec, 58°C for 1 min, 72°C for 2 min, with a final extension of 10 min at 72°C. The PCR product was purified using a Gel Purification Kit and cloned into the T/A cloning vector pGEM T-Easy (Promega). Positive clones of pT/A -357/+769 were isolated and sequenced.

### Luciferase-reporter plasmid constructs and assay

All NGX6 promoter fragments were cloned into pGL3 enhancer vector (Promega). Construct naming is based on the positions of the promoter fragments. Five deletion constructs of the NGX6 promoter region were created. These progressive deletion constructs (pGL3 -357/+769, pGL3 -357/+276, pGL3 +276/+769, pGL3 -357/-159, pGL3 -159/+276), originating from the construct pT/A -357/+769, were amplified by PCR using the primers listed in Table 1. All the primers included 9-bp non complementary extensions capable of generating KpnI or HindIII restriction sites. The promoter fragments were then subcloned into the KpnI/HindIII sites of pGL3 enhancer vector and sequenced.

**Table 1:** Primer pairs used for generating NGX6 promoter construct pGL3 -357/+769, pGL3-357/+276, pGL3 +276/+769, pGL3 -357/-159, pGL3 -159/+276.

pGL3 -357/+769	Forward: 5'-aaaggtaccCGAGCCCAGAGGGTTTACTT-3'
	Reverse: 5'-cccaagcttGCCTCAATCTTCCCTGCTTC-3'
pGL3 -357/+276	Forward: 5'-aaaggtaccCGAGCCCAGAGGGTTTACTT-3'
	Reverse: 5'-cccaagcttGGGGATTGGGATAGGACGAG-3'

pGL3 +276/+769	Forward: 5'-aaaggtaccCTCGTCCTATCCCAATCCCC-3'
	Reverse: 5'-cccaagcttGCCTCAATCTTCCCTGCTTC-3'

pGL3 -357/-159	Forward: 5'-aaaggtaccCGAGCCCAGAGGGTTTACTT-3'
	Reverse: 5'-cccaagcttCAAGCACAGAGCCCGAGGTC-3'

pGL3 -159/+276	Forward: 5'-aaaggtaccGGGGTGAGAAAGGCAGGGTC-3'
	Reverse: 5'-cccaagcttGGGGATTGGGATAGGACGAG-3'

Transfections were performed with Lipofectamine 2000 (Invitrogen, CarIsbad, CA); 5 × 10^5 ^cells were seeded in each well of 12-well tissue culture plate 24 h prior to transfection. The cells were transfected with 1 μg of various NGX6 promoter constructs, pGL3-control plasmid, or pGL3-enhancer plasmid, and 0.5 μg β-galactosidase vector for normalizing transfection efficiency per well according to manufacturer's instructions. Firefly luciferase activity was measured in cell lysates 48 h after transfection by using Luciferase Assay Kit (Promega). β-Galactosidase activity was measured in cell lysates by the β-galactosidase Enzyme Assay System (Promega). Experiments were repeated at least three times with three replicates per sample for each experiment. Results are normalized against β-galactosidase activity.

### Genomic DNA extraction and Bisulfite modification

Genomic DNA from cells and tissues was prepared using a DNA Extraction Kit (TaKaRa) according to the manufacturer's instructions. Five hundred nanograms of genomic DNA was modified and purified using an EZ DNA Methylation-Gold Kit (ZYMO REAEARCH), following the manufacturer's protocol. Modified DNA was used immediately or stored at -80°C for up to six months.

### Methylation-Specific PCR analysis of NGX6 promoter

The methylation-specific PCR primers were designed according to the promoter-active DNA sequence using Methyl Primer Express v1.0. Modified DNA was amplified by two different primer pairs specific to the unmethylated (u) and methylated (M) NGX6 promoter sequences, respectively. Methylation-specific primers (forward) 5'-AGGGATTAATCGAGTCGGTC-3', (reverse) 5'-ATAACCTCCGATATCCTCGC-3'. Unmethylation-specific primers (forward) 5'-GGTTTTATTGATAGGGATTA-3', (reverse) 5'-TATCCTCACAAACCCAAA-3'. PCR amplification was performed for a total of 38 cycles with an annealing temperature of 65°C and 55°C, respectively. Non-methylated and methylated human DKO DNA (ZYMO RESEARCH) was used as negative and positive control, respectively. Methylation-specific PCR products were analyzed by a 2% agarose gel and stained with ethidium bromide.

### NGX6 promoter methylation analysis by Sodium Bisulfite Sequencing

According to the result of CpGplot program, a 385-bp sequence which covers the whole sequence of MSP product was amplified. The primers were forward 5'-TGTGAGGATAGGGTTTTTTTGAGAT-3' and reverse 5'-CACCCCCRAAAAATAACCTC-3'. The PCR amplification was performed for a total of 38 cycles with annealing temperature of 60°C. PCR products were gel-purified and cloned into the T/A cloning vector pGEMT-Easy (Promega). Ten subclones were isolated and identified by double digestion and sequencing.

### 5-Aza-2'-deoxycytidine treatment

Human colon carcinoma cells HT-29 was grown for 5 days in the presence of various concentrations of 5-Aza-dC (0, 0.625, 1.25, 2.5, 5, and 10 μM). Fresh drug was add every 24 h. RNA and genomic DNA were separately isolated.

### Reverse transcription PCR and Real-time PCR

RNA was isolated from harvested cells with Trizol (Invitrogen) reagent and then treated with DNase (Roche) to eliminate possible contaminated DNA. Reverse transcription of the RNA was performed according to the instructions of Promega. 2 μl cDNA was used for each PCR using NGX6-special primers. The forward primer was 5'-AGAACCGCCATCCCTT-3', and the reverse primer was 5'-CACCTCGTGAGTCAAGCA-3'. The primers for GAPDH were as follows: forward, 5'-AGGTCGGAGTCAACGGATTTG-3'; reverse, 5'-GTGATGGCATGGACTGTGGT-3'. The GAPDH primers were added to the PCR at the end of the tenth cycle as control experiments. Ten microliters of each reaction was then run on 2% agarose gel and stained with ethidium bromide.

Then quantification of relative Quantification of relative transcript levels for NGX6 was performed using the BIO-RAD IQ™ 5 Muticolor Real Time PCR Detection System. cDNA samples were amplified with SYBR^® ^Premix Ex Taq™. The thermal cycling profile consisted of initial denaturation at 95°C for 30 s and 40 cycles at 95°C for 5 s, 54°C for 15 s, and 72°C for 30 s. Each sample was processed in triplicate. The expression level of NGX6 was normalized to GAPDH using IQ5 software.

### Statistical analysis of clinicopathological patient data

Statistical analyses were carried out using SPSS 16.0. Comparisons of categorical variables were made using χ^2 ^test or Fisher's exact test as appropriate. Differences were considered statistically significant when P-values were below 0.05.

## Results

### Cloning and bioinformatic analysis of the NGX6 5'upstream regulatory region

**Figure 1:**
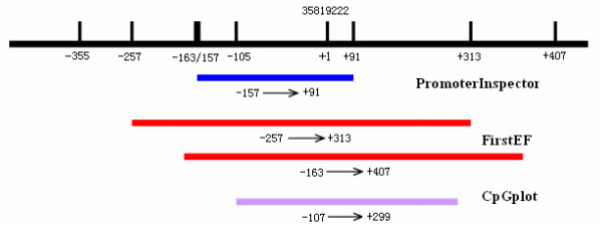
**Bioinformatics analysis of the NGX6 5' upstream regulatory region**. Schematic representation of the putative NGX6 promoter region and CpG island. The region showing the putative promoter activity and the CpG island are shown as a dark blue box, two red box, and a purple box, respectively. The genomic sequence number 35819222 of the NGX6 gene is defined as +1.

To clone the NGX6 promoter region, a database search against the human genomic DNA database using NGX6 (Genbank accession AF188239) as query http://www.ncbi.nlm.nih.gov/BLAST was performed to reveal the 5' upstream sequence of the NGX6 gene. Several bioinformatics tools were used to identify the potential promoter region of the NGX6 gene. A 248-bp region spanning positions -157 to +91 was identified as the potential promoter region of the NGX6 gene using PromoterInspector [11], whereas two regions located from -257 to +313 and -163 to +407 were identified as the NGX6 promoter using FirstEF program [12]. The 5' upstream sequence of the NGX6 gene was submitted for analysis to the CpGplot program [13]. Typical CpG islands were defined as ≥ 200 bp of sequence that had a C+G content of ≥ 50% and a value of >0.6 for the ratio (CpG observed)/(CpG expected) [14]. A CpG island that spanned positions -107 to +299 was detected using the program (Figure 1). Finally, a genomic DNA fragment that spanning positions -357 to +769 (the genomic sequence number 35819222 of the NGX6 gene is defined as +1) was amplified by PCR. The fragment was then cloned into the T/A cloning vector pGEM-T-Easy (Promega).

### Identification of NGX6 proximal promoter

In order to identify the proximal promoter region of NGX6 gene, four constructs of progressive deletion were generated and cloned to the upstream of the luciferase reporter gene in the pGL3-enhancer vector (Promega). Transient transfection experiments were carried out using promoter deletion constructs spanning positions -357 to +769. Plasmid pGL3-357/+276, pGL3+276/+769, pGL3 -357/-159 and pGL3-159/+276 were transfected with Lipofectamine 2000 (Invitrogen) into Cos7 and HT-29 cells, respectively. The luciferase activity driven by NGX6 promoter constructs was measured 48 h after transfection. Expression levels were corrected for variable transfection efficiencies by cotransfection with a plasmid directing the β-galactosidase expression.

**Figure 2:**
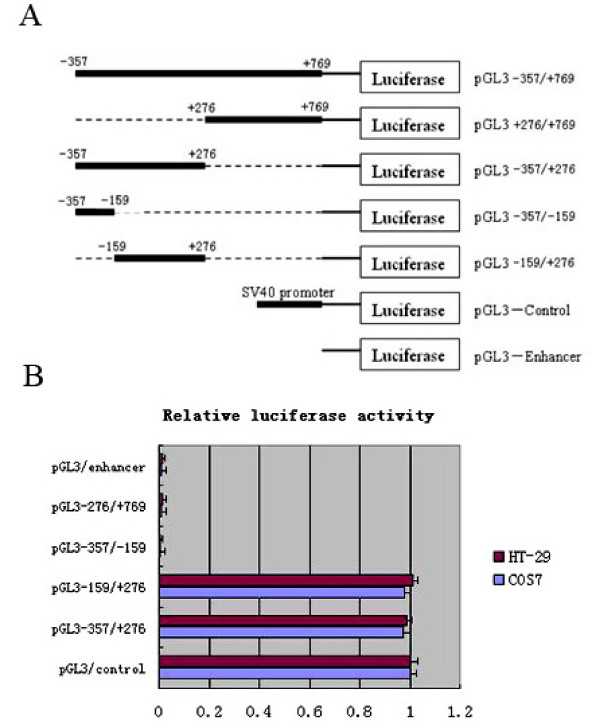
**Deletion analysis of the NGX6 promoter**. (A) Schematic illustration of deletion constructs of NGX6 promoter. (B)Luciferase activity of the deleted constructs in Cos7 and HT-29 cells. PGL3-enhancer, PGL3-control and NGX6-promoter deleted constructs were transfected into Cos7 and HT-29 cells. All of the constructs were cotransfected with β-galactosidase vector for normalizing transfection efficiency. Data are the meas ± SD of three independent experiments. All the transfection experiments were repeated at least three times.

As shown in Figure 2, in Cos7 and HT-29 cells, the reporters driven by the shorter 433-bp fragment (pGL3-157/+276) which should contain the core promoter, showed as high luciferase expression as the SV40 promoter of pGL3-control. Luciferase expression driven by the construct pGL3 +276/+769 and pGL3 -357/-159 exhibited as extremely low as pGL3-enhancer. Thus, the sequence spanning positions -159 to +276 was identified as a promoter. MethPrimer Program analysis showed that the NGX6 promoter region had high G/C content and characteristic of a CpG island [15]. In addition, no canonical TATA boxes, but two CAAT boxes were found in this promoter region by the MatInspector program (data not shown).

### Frequent aberrant methylation of NGX6 promoter in colorectal cancer cell lines and tissues

**Figure 3:**
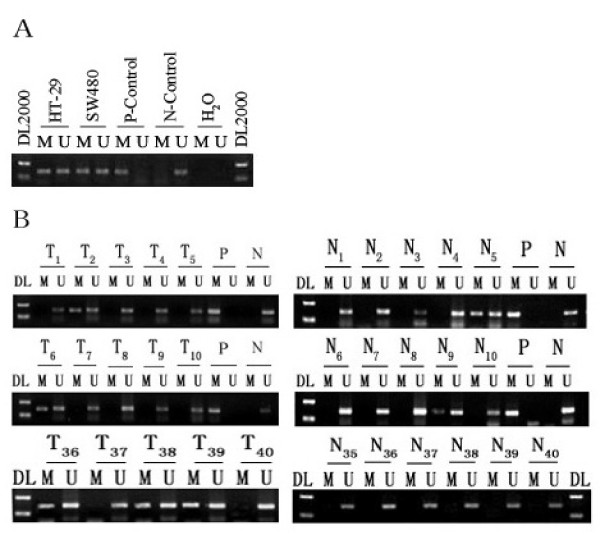
**Methylation of the NGX6 promoter in colorectal cancer**. (A) Methylation of the NGX6 promoter in colon cancer cells HT-29 and SW480. (B) DNA from 40 colorectal cancer tissue (T) and 40 adjacent normal colorectal tissues (N) were bisulfite-modified and analyzed for methylaion of the NGX6 promoter by methylation-specific PCR analysis. "DL": DNA marker2000; "P": positive methylated control; "N": negative unmethylated control; "M": methylated NGX6; "U": unmetylated NGX6.

Epigenetic modification is a frequent mechanism of inactivation of tumor suppressor genes in colorectal cancer [16]. To clarify the mechanism of NGX6 gene inactivation in colorectal cancer cells and tissues, methylation-specific PCR was used to examine the methylation status of the NGX6 promoter. Both HT-29 and SW480 cell lines showed methylation of the NGX6 promoter (Figure 3A).

To identify whether aberrant methylation of the NGX6 promoter in colon cancer cell lines reflects an epigenetic event occurring in primary colorectal cancer tissues, we further examined the NGX6 promoter mathylation status in the 40 pairs of colorectal cancers and adjacent normal colorectal mucosa using methylation-specific PCR (some are shown in Figure 3B). The methylated PCR product was detected in 18/40 (45%) cancer samples and 7/40 (17.5%) adjacent normal samples (Table 2).

**Table 2:** NGX6 promoter methylation frequency in colorectal cancer and adjacent normal mucosa.

		Methylation status	
		
variable	n	Absent	Present
Colorectal cancer	40	22 (55%)	18 (45%)

Adjacent normal mucosa	40	33 (82.5%)	7 (17.5%)

To clarify whether the NGX6 methylation status of the colorectal samples was correlated with clinicopathological features of colorectal cancer patients, univariate analyses were carried out to correlate the methylation status of the NGX6 promoter with various clinicopathological parameters. As shown in Table 3, only methylation of the NGX6 promoter was found to correlate with age in colorectal cancer (p < 0.05).

**Table 3:** Clinicopathological parameters compared to NGX6 promoter methylation analyed by Chi-square Test or Fisher' s Exact Test.

	Methylation status		
		
Variable	Absent	Present	*P*-value
Sex			

Male	9(47.4%)	10(52.6%)	0.369
	
Female	13(61.9%)	8(38.1%)	

Age			

<65 Years	11(40.7%)	16(59.3%)	0.008
	
≥ 65 Years	11(84.6%)	2(15.4%)	

Dukes stage			

A	4(66.7%)	2(33.3%)	0.111
	
B	10(71.4%)	4(28.6%)	
	
C	5(38.5%)	8(61.5%)	
	
D	3(42.9%)	4(57.1%)	

Metastasis status			

Negative	14(70.0%)	6(30.0%)	0.056
	
Positive	8(40.0%)	12(60.0%)	

Primary site			

Colon	7(38.9%)	11(61.1%)	0.067
	
Rectum	15(68.2%)	7(31.8%)	

**Figure 4:**
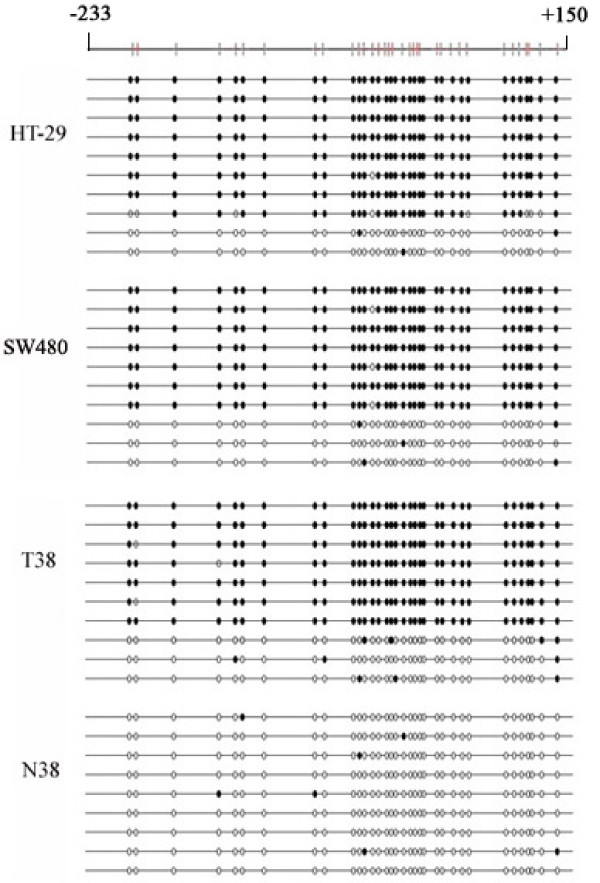
**Bisulfite sequence analysis of NGX6 promoter**. Methylation status of CpG sites around the NGX6 promoter region were analyzed in colon cancer cell lines HT-29 and SW480, colon cancer tissue (T38) and adjacent normal colorectal tissues (N38). Region spans -233 to +150 including 34 CpG sites. Each row represents an individual subclone. White circles represent unmethylated CpGs. Black circles represent methylated CpGs.

To determine a more detailed map of the methylation in the NGX6 promoter, we performed bisulfite sequencing around the promoter region of the NGX6 gene in colon cancer cell lines and some of the colorectal cancer. Specific primers without CpG sites were used to amplify the region spanning position -233 to +150. The sequence including 34 CpG sites was showed in Figure 4. Bisulfite sequencing of 10 individual clones of PCR products from primary colon cancer biopsies (T38) and cell lines (HT-29 and sw480) revealed densely methylated CpGs within the promoter region compared to normal colon tissue (N38).

### 5'-Aza-dC augmented endogenous mRNA and reversed the methylation status of NGX6 promoter in colon cancer cells

**Figure 5:**
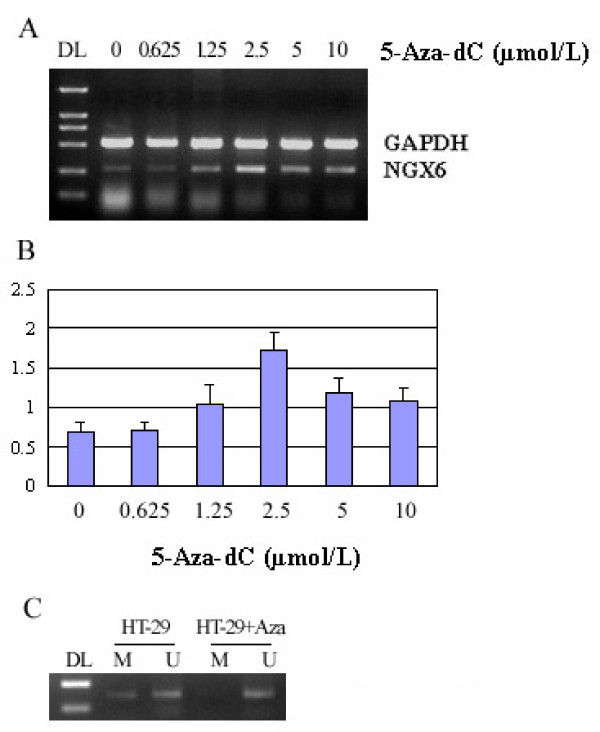
**5-Aza-dC induced the expression of NGX6 in HT-29 cells**. (A) Colon cancer cell line HT-29 was treated with six different doses of 5-Aza-dC (0.625, 1.25, 2.5, 5.0 and 10 μM), as indicated above each lane, for five days. Total RNA was isolated from control, and treated cells were then analyzed by RT-PCR. (B) NGX6 mRNA level was analyzed by real-time PCR after 5-Aza-dC treament. (C). colon cancer cell line HT-29 was treated with 2.5 μM 5-Aza-dC for five days. DNA was isolated and bisulfite-modified, and then analyzed for methylaion of the NGX6 promoter by methylation-specific PCR analysis. "DL": DNA marker 2000; "M": methylated NGX6; "U": unmetylated NGX6.

In order to clarify the functional association between NGX6 promoter methylation and its aberrant expression in CRC, a DNA demethylating agent, 5-Aza-dC was used to treat HT-29 cells. After 5 days treatment, we isolated mRNA and genomic DNA from the cells, and then detected NGX6 mRNA re-expression in these cells using RT-PCR assay (Figure 5A) and Real-time PCR (Figure 5B). NGX6 expression increased with increasing dosage of 5-Aza-dC. NGX6 expression was higher when induced by 2.5 μM 5-Aza-dC. To confirm that reactivation of NGX6 mRNA expression in HT-29 cell line was caused by demethylation of the NGX6 promoter, methylation-specific PCR was used to detect methylation status changes in the NGX6 promoter in HT-29 cell lines after 5-Aza-dC treatment. The results showed that 2.5 μM 5-Aza-dC could reverse the methylation of NGX6 promoter in HT-29 cells (Figure 5C).

## Discussion

As a candidate tumor suppressor gene, down-regulation of the NGX6 gene has been shown to be critical to the pathogenesis of colorectal cancer [17,18]. However, the mechanism responsible for the inactivation of NGX6 gene in colorectal cancer has not been investigated. To delineate the control mechanisms of NGX6 gene expression, we reported, for the first time, the cloning and functional characterization of the NGX6 promoter.

In this study, we cloned and characterized 1126 bp (-357 to +769) of the 5' genomic region of NGX6 gene, which exhibits promoter activity as SV40 promoter. In order to define the proximate regulatory regions in the NGX6 promoter, we generated a series of 5'-deleted constructs and transfected into Cos7 and HT-29 cells, respectively. The results of luciferase expression indicated that a 433-bp region (-157 to +276) was found to be necessary for the transcriptional activity of the NGX6 gene, which suggested that we have found a function of the NGX6 promoter.

As the result of bioinformational analysis, the functional NGX6 promoter region is a TATA-less, GC-rich promoters and has several CAAT boxes. Meanwhile, there is a CpG island in the region of NGX6 promoter, and its function has remained undetermined. It is well known that aberrant promoter hypermethylation is thought to contribute to carcinogenesis by inactivating tumor suppressor genes. Many tumor suppressor genes silencing described in colorectal cancers have been linked to promoter hypermethylation such as p16, MLH1, TSP-1 and APC [19-22]. Here, the NGX6 promoter was found to be methylated in colon cancer cell line HT-29.

The prevalence of NGX6 promoter methylation in colorectal cancers was 45% (18/40) compared with 17.5% (7/40) in normal colorectal mucosa (P < 0.008). This result suggested that the aberrant methylation of the NGX6 gene was frequent in colorectal cancers. Then the correlation between the methylation status and the clinicopathological findings in 40 colorectal cancer tissues was evaluated. In the study, a significant difference was observed in age (p = 0.008). Moreover, a trend was shown toward metastasis status and primary site in colorectal carcinomas with NGX6 promoter methylation (p = 0.056 and P = 0.067, respectively). And bisulfite sequence analysis of the CpG islands around NGX6 promoter indicate that dense methylation of CpG sites in colon cancer cell lines and colorectal cancer tissues compared with normal colorectal tissues. NGX6 promoter methylation may serve as a biomarker for diagnosis. Since the ability of invasion and metastasis is closely related to prognosis in colorectal cancer, NGX6 methylation may also be a biomarker for prognosis. Of course, all of these hypotheses must be further studied using a large number sample analysis.

Treatment of HT-29 cells with 5-Aza-dC restored NGX6 expression suggests that aberrant hypermethylation of the promoter is directly responsible for ranscription inactivation of its expression in colon cancer cells. These methylated CpG dinucleotides lie in regions that can inhibit NGX6 gene transcription through one of two mechanisms. First, transcription factor binding may be inhibited by methylated CpG dinucleotides. And second, proteins that recognize methyl-CpG can elicit the repressive potential of methylated DNA [23]. The methylase inhibitor can reverse NGX6 expression in colorectal cancer, and it is possible to restore its function as tumor suppressor gene at some a degree. This shows that NGX6 may be a potential target for therapy.

## Conclusions

In summary, through methylation-specific PCR, bisulfite sequencing and demethylation treatment, we have established that loss of NGX6 expression in colorectal cancers may be due in part to methylation of CpG sites within the NGX6 promoter. NGX6 promoter methylation may be used as a potential biomarker for diagnosis and prognosis, or a useful target for therapy.

## Competing interests

The authors declare that they have no competing interests.

## Authors' contributions

MJL participated in the study design and coordination, data collection, drafting of the manuscript and DNA methylation analysis. YP and QG Contributed to patient recruitment, obtaining consent, surgical sample collection and handing, gDNA extraction and DNA methylation analysis. XYW participated in experimental design, helped to draft the manuscript and carried out data interpretation. SRS and GYL carried out the experiment design, manuscript drafting and revision.

All authors read and approved the final manuscript.

## Pre-publication history

The pre-publication history for this paper can be accessed here:

http://www.biomedcentral.com/1471-2407/10/160/prepub

## References

[B1] Jones PA Baylin SB the epigenomics of cancer Cell 2007 128 4 683 692 17320506 10.1016/j.cell.2007.01.029 PMC3894624

[B2] Jubb AM Bell SM Quirke P Methylation and colorectal cancer J Pathol 2001 195 111 134 11568897 10.1002/path.923

[B3] Yang JB Bin LH Li ZH Zhang XH Qian J Zhang BC Zhou M Xie Y Deng LW Li GY Refined localization and cloning of a novel putative tumor suppressor gene associated with nasopharyngeal carcinoma on chromosome 9p21-22 Clin J Cancer 2000 19 1 6 9

[B4] Fan SQ Zhang WL Peng SP Zhou M Li GY Study of in situ expression of NGX6 gene in the several common types of cancer and its clinical significance Progress in Biochemistry and Biophysics 2008 35 9 1014 1020

[B5] Zhang XM Sheng SR Wang XY Wang JR Li J Expression of tumor relatedgenes NGX6 in gastric and colorectal cancer World Chinese Journal of Digestology 2002 10 8 873 876

[B6] Wang XY Shen SR Liu HY Zhang XM Peng C Huang H Liu F Li XL Li GY Effects of tumor suppressor gene NGX6 on growth of human colon cancer cell line HT-29 World Chin J Digestol 2004 12 3 574 579

[B7] Xiao ZM Shen SR Lian P Intraspleenic tumor modle of nude mice in the anti-metastasis roles of NGX6 gene against colon cancer Journal of Central South University (Medical Sciences) 2007 32 5 753 757 18007065

[B8] Liu F Shen SR Li HT Wang XY Peng Y Liao MT Guo Q Effects of NGX6 on the transcriptional activation of beta-catenin/TCF/LEF in Wnt/beta-catenin signal pathway Journal of Central South University (Medical Sciences) 2007 32 6 985 991 18182714

[B9] Wang XY Shen SR Liu F Peng Y Li GY Fan SQ Inhibitory effects of NGX6 gene on EGFR/K-r as/JNK/c-Jun/cyclin D1 signal pathway in the colon cancer Progress in Biochemistry and Biophysics 2008 35 5 570 576

[B10] Peng Y Li HT Wu MH Wang XY Fan SQ Liu F Xiang B Guo Q Tang XY Shen SR NGX6 inhibits AP-1 and Ets-1 expression and down-regulates cyclin D1 in human colorectal cancer Acta Biochim Biophys Sin 2009 41 6 504 514 19499154 10.1093/abbs/gmp039

[B11] Genomatix GEMlauncher PromoterInspector Search for the mammalian promoters http://www.genomatix.de/cgi-bin/promoterinspector_prof/promoterinspector.pl

[B12] FirstEF: first-exon and promoter prediction program for human DNA http://rulai.cshl.org/tools/FirstEF/

[B13] European Bioinformatic Institute CpGPlot program http://www.ebi.ac.uk/emboss/cpgplot

[B14] Gardiner-Garden M Frommer M CpG islands in vertebrate genomes J Mol Biol 1987 196 2 261 282 3656447 10.1016/0022-2836(87)90689-9

[B15] University Bioinformatic Institute MethPrimer-Design Primers for Methylation PCRs http://www.urogene.org/methprimer/

[B16] Jubb AM Bell SM Quirke P Methylation and colorectal cancer J Pathol 2001 195 111 134 11568897 10.1002/path.923

[B17] Wang XY Shen SR Liu HY Li XL Fan SQ Effects of NGX6 Gene on Cell Cycle in Colon Cancer Cell Line HT-29 Progress in Biochemistry and Biophysics 2006 33 1 45 50

[B18] Lian P Guo Q Peng Y Xiao ZM Liu F Wang XY Shen SR Li GY The Role of NGX6 Gene on Apoptosis of Human Colon Cancer Progress in Biochemistry and Biophysics 2008 35 10 1154 1160

[B19] Kim HC Lee HJ Roh SA Kim JS Yu CS Kim JC CpG island methylation in familial colorectal cancer patients not fulfilling the Amsterdam criteria J Korean Med Sci 2008 23 2 270 277 18437011 10.3346/jkms.2008.23.2.270 PMC2526421

[B20] Vilkin A Niv Y Nagasaka T Morgenstern S Levi Z Fireman Z Fuerst F Goel A Boland CR Microsatellite instability, MLH1 promoter methylation, and BRAF mutation analysis in sporadic colorectal cancers of different ethnic groups in Israel Cancer 2009 115 4 760 769 19127559 10.1002/cncr.24019 PMC2855188

[B21] Kumar K Brim H Giardiello F Nouraie M Lee EL Ashktorab H Distinct BRAF (V600E) and KRAS mutations in high microsatellite instability sporadic colorectal cancer in African Americans Clin Cancer Res 2009 15 4 1155 1161 19190129 10.1158/1078-0432.CCR-08-1029 PMC2713502

[B22] Yi JM Tsai HC Glöckner SC Lin S Ohm JE Easwaran H James CD Costello JF Riggins G Eberhart CG Laterra J Vescovi AL Ahuja N Herman JG Schuebel KE Baylin SB Abnormal DNA methylation of CD133 in colorectal and glioblastoma tumors Cancer Res 2008 68 19 8094 8103 18829568 10.1158/0008-5472.CAN-07-6208 PMC2744404

[B23] Klose RJ Bird AP Genomic DNA methylation: the mark and its mediators Trends Biochem Sci 2006 31 2 89 97 16403636 10.1016/j.tibs.2005.12.008

